# Clinical outcomes of hospitalised patients with catheter-associated urinary tract infection in countries with a high rate of multidrug-resistance: the COMBACTE-MAGNET RESCUING study

**DOI:** 10.1186/s13756-019-0656-6

**Published:** 2019-12-03

**Authors:** Aina Gomila, Jordi Carratalà, Noa Eliakim-Raz, Evelyn Shaw, Cristian Tebé, Martin Wolkewitz, Irith Wiegand, Sally Grier, Christiane Vank, Nienke Cuperus, Leonard Van den Heuvel, Cuong Vuong, Alasdair MacGowan, Leonard Leibovici, Ibironke Addy, Miquel Pujol, on behalf of RESCUING Study Group and Study Sites

**Affiliations:** 1 Department of Infectious Diseases, Hospital Universitari de Bellvitge-Institut d’Investigació Biomèdica de Bellvitge (IDIBELL), Institut Català de la Salut (ICS-HUB), Barcelona, Spain; 2 Corporació Sanitària Parc Taulí, Barcelona, Spain; 3 Spanish Network for Research in Infectious Diseases (REIPI RD12/0015), Instituto de Salud Carlos III (ISCIII), Madrid, Spain; 4 University of Barcelona, Barcelona, Spain; 5 Department of Medicine E, Beilinson Hospital, Rabin Medical Center, Petah-Tiqva; and Sackler Faculty of Medicine, Tel-Aviv University, Tel-Aviv, Israel; 6 Institut d’Investigació Biomèdica de Bellvitge (IDIBELL) and Department of Basic Sciences, Universitat Rovira i Virgili, Tarragona, Spain; 7 Institute of Medical Biometry and Statistics, University of Freiburg, Freiburg, Germany; 8 AiCuris Anti-infective Cures GmbH, Wuppertal, Germany; 9 Department of Medical Microbiology, Southmead Hospital, North Bristol NHS Trust, Bristol, UK; 10 Julius Center for Health Sciences and Primary Care, University Medical Center Utrecht, Utrecht, Netherlands

**Keywords:** Catheter-associated urinary tract infection, Antimicrobial resistance, Gram-negative infections

## Abstract

**Background:**

Although catheter-associated urinary tract infection (CA-UTI) is a major healthcare-related problem worldwide, there is a scarcity of current data from countries with high antimicrobial resistance rates. We aimed to determine the clinical outcomes of patients with CA-UTI compared to those of patients with other sources of complicated urinary tract infection (cUTI), and to assess the impact of antimicrobial resistance. We also aimed to identify the factors influencing 30-day mortality among patients with CA-UTI.

**Methods:**

This was a multicentre, multinational retrospective cohort study including hospitalised adults with cUTI between January 2013 and December 2014 in twenty hospitals from eight countries from southern Europe, Turkey and Israel. The primary endpoint was 30-day mortality. The secondary endpoints were length of hospital stay, symptom improvement after 7 days’ treatment, symptom recurrence at 30 days and readmission 60 days after hospital discharge.

**Results:**

Of the 807 cUTI episodes, 341 (42.2%) were CA-UTIs. The time from catheter insertion to cUTI diagnosis was less than 2 weeks in 44.6% of cases. Overall, 74.5% of cases had hospital or healthcare-acquired CA-UTI. Compared to patients with other cUTI aetiologies, those with CA-UTI had the following characteristics: they were more frequently males, older, admitted for a reason other than cUTI and admitted from a long-term care facility; had higher Charlson’s comorbidity index; and more frequently had polymicrobial infections and multidrug-resistant Gram-negative bacteria (MDR-GNB). Patients with CA-UTI also had significantly higher 30-day mortality rates (15.2% vs 6%) and longer hospital stay (median 14 [interquartile range -IQR- 7-27] days vs 8 [IQR 5–14] days) than patients with cUTI of other sources. After adjusting for confounders, CA-UTI was not independently associated with an increased risk of mortality (odds ratio, 1.40; 95% confidence interval, 0.77–2.54), and neither was the presence of MDR-GNB.

**Conclusions:**

CA-UTI was the most frequent source of cUTI, affecting mainly frail patients. The mortality of patients with CA-UTI was high, though this was not directly related to the infection.

## Introduction

Urinary tract infection (UTI) is a major healthcare problem worldwide. Catheter-associated UTI (CA-UTI) accounts for more than 1 million cases per year in the US [1]. These infections are also responsible for more than 80% of UTIs originating in the healthcare setting and are the most frequent cause of both bacteraemia in long-term care facilities (LTCF) and infection in patients with spinal cord injury [2, 3]. Approximately 20% of hospitalised patients have a urinary catheter during admission, with the risk of CA-UTI increasing by 3–7% per day [4, 5].

Despite efforts to reduce the incidence of CA-UTI, rates continue to increase, with the US Centres for Disease Control and Prevention reporting a 6% increase between 2009 and 2013 [6]. The high prevalence of CA-UTI is associated with significant antimicrobial use, and this not only favours the emergence and spread of multidrug-resistance (MDR) but also increases healthcare costs [7–9]. Antibiotic resistance, in particular, has become a major healthcare problem that complicates treatment and leads to worse outcomes. Even though these issues exist, CA-UTI is usually seen as a mild or low-risk infection with no severe consequences for patients. However, most previous studies seeking to address this issue have only evaluated specific populations, such as critically ill patients, which limits extrapolation to other contexts. Thus, the question of whether CA-UTI development increases the risk of mortality remains unanswered, not least because conflicting results have previously been reported [10, 11].

We aimed to assess the clinical outcomes of CA-UTI among hospitalised patients with complicated UTI (cUTI) in a multicentre study of surgical and medical patients from eight countries with high prevalence of multidrug-resistant Gram-negative bacteria (MDR-GNB).

## Methods

### Study design

The COMBACTE-MAGNET WP5 RESCUING study is an international, multi-centre, retrospective, observational cohort study conducted between January 2015 and August 2016. It included both patients diagnosed with cUTI as the primary reason for hospitalisation and those who developed cUTI after being hospitalised for another reason between 1st January 2013 and 31st December 2014 [12, 13]. The STROBE guidelines for reporting observational studies were followed [14]. In the present study, we compared patients who had CA-UTI with those who had cUTI of other sources.

### Setting and patients

The study was conducted at 20 hospitals in Bulgaria, Greece, Hungary, Israel, Italy, Romania, Spain and Turkey. Patient selection was done by searching the appropriate Clinical Modification codes at discharge based on versions 9 or 10 of the International Classification of Diseases. The sample size was calculated to detect an absolute difference of 10% in the treatment failure rate between infection due to MDR and other pathogens (power = 0.83, α = 0.05). We planned to include 50 to 60 patients per hospital to reach a total estimated sample size of 1000 [15, 16].

The inclusion criteria were based on the Food and Drug Administration guidance for cUTI [17], requiring patients to have a UTI plus at least one of the following:

1. At least one from among the following: an indwelling urinary catheter; urinary retention (at least 100 mL of residual urine after voiding); neurogenic bladder; obstructive uropathy (e.g., nephrolithiasis or fibrosis); renal impairment caused by intrinsic renal disease (estimated glomerular filtration rate < 60 mL/min); and renal transplantation; urinary tract modification (ileal loop or pouch).

2. At least one from among the following signs or symptoms: Chills or rigors associated with fever or hypothermia (temperature > 38 °C or < 36 °C); flank or pelvic pain, dysuria, urinary frequency or urgency; and costovertebral angle tenderness on physical examination.

3. A urine culture with at least 10^5^ colony-forming units (CFU)/mL of a uropathogen (no more than two species); or at least one blood culture growing possible uropathogens (no more than two species) with no other evident site of infection.

We excluded patients who were younger than 18 years, diagnosed with prostatitis (based on Food and Drug Administration guidance), diagnosed with pyelonephritis with normal urinary tract, had polymicrobial infections that included Candida spp. or more than two bacterial species, or who had cUTI with Candida spp. as the sole uropathogen.

### Data collection

Data from eligible patients were collected from January 2015 to August 2016. For all patients, a standardised data set was collected retrospectively from electronic hospital records and input in a web-based electronic case report form (eCRF) with controlled access. The data set included details of demographic characteristics, comorbidities, infection acquisition site, signs and symptoms, laboratory and microbiology test results, and discharge and outcome details, including death. The follow-up period was limited to two months after hospital discharge. To ensure data quality, study sites were monitored and audited.

### Definitions

Acquisition of cUTI in a medical care facility was considered hospital-acquired if it started 48 h or more after hospital admission. Acquisition of cUTI was considered healthcare-associated if it was detected at hospital admission or within the first 48 h of hospitalisation, and met any of the following criteria: the patient had received intravenous therapy, wound care or specialist nursing care at home in the previous 30 days; hospital or haemodialysis ward attendance or intravenous chemotherapy administration in the previous 30 days; hospitalisation for at least 2 of the previous 90 days; residence in a long-term care facility; underwent an invasive urinary procedure in the previous 30 days; or had a long-term indwelling urethral catheter.

We then defined cUTI as either CA-UTI or cUTI of other source (other-cUTI). The CA-UTI group comprised those with UTI related to indwelling urinary catheterisation, including long-term, short-term or intermittent catheterisation. The other-cUTI group included those with all other causes of cUTI, including the following: UTI related to anatomical urinary tract modification (including any urinary diversion procedure, nephrostomy, stent or renal transplant); UTI related to obstructive uropathy (including any obstruction intrinsic or extrinsic to the urinary tract, such as lithiasis, tumour, ureteral herniation or prostate hyperplasia); UTI related to events that do not fall under any other category (such as neurogenic bladder).

MDR was defined in accordance with the international expert proposals of Magiorakos et al., as non-susceptibility to at least one agent in three or more antimicrobial categories (extended-spectrum penicillins, carbapenems, cephalosporins, aminoglycosides and fluoroquinolones) [18]. Extensive drug-resistance (XDR) was defined as non-susceptibility to at least one agent in all but two or fewer antimicrobial categories.

Steroid therapy was defined as the administration of a dose of at least 10 mg of prednisolone or an equivalent dose of another steroid for 30 days or more prior to the diagnosis of cUTI.

Length of hospital stay was considered in all cases from the day of diagnosis of cUTI to the day of discharge or mortality.

### Clinical outcomes

The primary endpoint was 30-day mortality. The secondary endpoints were length of hospital stay, symptom improvement at 7 days of treatment, symptom recurrence at 30 days from diagnosis, and readmission rate at 60 days from discharge. We also aimed to identify the factors influencing 30-day mortality among patients with CA-UTI.

### Statistical methods

Demographic, clinical and outcome data for patients in the CA-UTI and other-cUTI groups were described using appropriate statistics according to the nature and distribution of the variable. The statistical analyses were performed using version 3.5.0 of R for Windows. Statistical significance was set at a probability level of < 0.05.

The crude and adjusted association between the presence of CA-UTI and the 30-day mortality was analysed by logistic modelling with mixed-effects that took into account the variability between centres. The patient demographics and variables associated with 30-day mortality by unadjusted analysis (Charlson index, having haematological malignancy, basal functional status, place of cUTI acquisition and reason for admission) were used for adjustment. Although these adjustment variables were initially chosen on clinical grounds, they were required to modify the coefficient of the main variable (CA-UTI) by more than 10 to remain in the model. The impact of the assessment centre was evaluated by the intra-class correlation (ICC), which measures how much of the overall variation in the outcome is explained simply by clustering. ICC ranges from 0 to 1; a value close to 1 indicates that patients within centers are more similar than patients between centers, and a value close to 0 indicates that patients between centers are similar.

Residues were validated graphically, and the conditions of application of the models were tested. The odds ratios (ORs) and 95% confidence intervals (CIs) were calculated when appropriate.

## Results

### Baseline patient characteristics

Table 1 shows the baseline characteristics of patients in the CA-UTI and other-cUTI groups. In total, 807 episodes of cUTI were included, of which 341 (42.2%) had CA-UTIs. The time between catheter insertion and diagnosis was < 2 weeks in 44.6%. The CA-UTI was hospital-acquired in 130 (38.1%), healthcare-acquired in 124 (36.4%) and community-acquired in 87 (25.5%) cases. Among those with hospital-acquired disease, 50 (38.4%) cases had onsets in ICU. Compared with the other-cUTI group, the CA-UTI group were more frequently male, older, admitted for a reason other than cUTI, and acquired the infection in a medical care facility. The Charlson score was also higher in the CA-UTI group.

**Table 1:** Baseline characteristics of patients with CA-UTI and other-cUTI (*n* = 807 episodes)

Factors	CA-UTI (*n* = 341)	Other cUTI (*n* = 466)	*p*-value
Male gender, *n* (%)	197 (57.8%)	222 (47.6)	0.006
Age, median (IQR)	71 (60–82)	68 (56.2–79)	0.009
Diabetes mellitus, *n* (%)	109 (32.2)	113 (24.2)	0.019
Haematological malignancy, *n* (%)	8 (2.3)	10 (2.1)	0.84
Solid tumour, *n* (%)	42 (12.3)	63 (13.8)	0.74
Liver disease, *n* (%)	16 (4.7)	26 (5.58)	0.68
Urgent admission, *n* (%)	285 (83.6)	394 (84.5)	0.78
Admission reason:			< 0.001
Other reasons	213 (62.5)	120 (25.8)	
cUTI	128 (37.5)	346 (74.2)	
Admission from medical care facility, *n* (%)	88 (25.8)	90 (13.5)	< 0.001
Charlson score, median (IQR)	3 (1–5)	2 (0–4)	< 0.001
Organ transplantation, *n* (%)	10 (2.9)	50 (10.7)	< 0.001
Immunosuppressive therapy, *n* (%)	19 (5.6)	63 (13.5)	< 0.001
Steroid therapy, *n* (%)	22 (6.5)	32 (6.87)	0.92
Functional capacity: Bedridden, *n* (%)	91 (26.7)	55 (11.8)	< 0.001
Chronic renal impairment, *n* (%)	72 (21.2)	141 (30.8)	0.005
UTI within 1 year, *n* (%)	79 (23.2)	143 (30.8)	0.021
Antibiotic within 30 days, *n* (%)	67 (19.7)	104 (22.5)	0.39
Acquisition of cUTI at a medical care facility, *n* (%)	254 (74.5)	160 (34.3)	< 0.001
Urinary retention, *n* (%)	70 (20.5)	112 (24)	0.27
Neurogenic bladder	32 (9.4)	12 (2.58)	< 0.001
Obstructive uropathy	43 (12.6)	166 (35.8)	< 0.001
Severity of infection: Severe sepsis/septic shock, *n* (%)	49 (15.4)	70 (15.9)	0.92

*CA-UTI* catheter-associated urinary tract infection (CA-UTI), complicated urinary tract infection due to other sources (other-cUTI). *IQR* Interquartile range, *cUTI* complicated urinary tract infection, and *UTI* urinary tract infection

### Causative agents

Table 2 shows the most frequent aetiologies of cUTI. Compared with the other-cUTI group, the CA-UTI group more frequently had polymicrobial infection (21.1% vs 10.1%, *p* < 0.001), infections caused by *Pseudomonas aeruginosa* (16.7% vs 7.7%, p < 0.001), *Proteus mirabilis* (11.7% vs 5.6%, *p* < 0.001) or Enterococcus spp. (11.1% vs 6.4%, *p* = 0.017). There was also a higher frequency of MDR-GNB in the CA-UTI group compared with the other-cUTI group (35.2% vs 23%, *p* < 0.001). The MDR-GNB resistance profiles in patients with CA-UTI are shown in the Additional file 1.

**Table 2:** Causative agents for CA-UTI and other-cUTI

Causative agents	CA-UTI (*n* = 341)	Other cUTI (*n* = 466)	*p*
Bacteraemia, *n* (%)	62 (18.2)	67 (14.4)	0.14
Polymicrobial infection, *n* (%)	72 (21.1)	47 (10.1)	< 0.001
*Escherichia coli*, *n* (%)	127 (37.2)	287 (61.6)	< 0.001
*Klebsiella pneumoniae*, *n* (%)	63 (18.5)	80 (17.2)	0.63
*Pseudomonas aeruginosa*, *n* (%)	57 (16.7)	36 (7.7)	< 0.001
*Proteus mirabilis, n (%)*	40 (11.7)	26 (5.6)	0.002
*Acinetobacter baumannii, n (%)*	22 (6.5)	3 (0.6)	< 0.001
MDR-GNB, *n* (%)	120 (35.2)	107 (23)	< 0.001
Enterococcus, *n* (%)	38 (11.1)	30 (6.4)	0.017
*Staphylococcus aureus*, *n* (%)	6 (1.8)	4 (0.9)	0.25
Other microorganisms, *n* (%)	55 (16.1)	45 (9.7)	0.006

*CA-UTI* catheter-associated urinary tract infection, complicated urinary tract infection due to other sources (other-cUTI), and *MDR-GNB* multidrug-resistant Gram-negative bacteria

### Clinical outcomes

#### Primary outcome

The 30-day mortality in the CA-UTI group was significantly higher than that in the other-cUTI group (52/341 [15.2%] vs 28/466 [6%], *p* < 0.001). Table 3 shows the crude association between patient characteristics and 30-day mortality considering the hospital effect in the entire cohort. Even though ICC values are not far from 0, values above 0.20 suggest that patients’ characteristics within centers are more similar than patients’ characteristics between centers. The unadjusted OR of CA-UTI for 30-day mortality was 2.56 (95% CI 1.52–4.32). Table 4 shows the adjusted models of mortality first in the entire cohort (Model 1), secondly in the subgroup of patients admitted for cUTI (Model 2) and thirdly in patients admitted for other conditions than cUTI but who developed cUTI during hospitalisation (Model 3). To evaluate the effect of CA-UTI on 30-day mortality, the factor *presence of severe sepsis or septic shock* (unadjusted OR 11.7) was not included in the models because its strong association precluded the evaluation of any other effects. After adjustment, in none of the three models the CA-UTI remained as independent predictor of 30-day mortality (OR 1.40; 95%CI 0.77–2.54 in Model 1, OR 1.62; 95% CI 0.63–4.15 in Model 2 and OR 1.24; 95% CI 0.54–2.81 in Model 3).

**Table 3:** Crude association between the baseline characteristics of patients with cUTI and the 30-day mortality

Variable	OR	95% CI	*p*-value	ICC^a^
Male gender	0.62	0.39–1.01	0.07	0.2039
Age	1.03	1.01–1.05	0.001	0.1871
Diabetes mellitus	0.98	0.58–1.66	0.82	0.1989
Haematological malignancy	6.02	2.11–17.23	< 0.001	0.1918
Solid tumour	1.5	0.78–2.89	0.36	0.2035
Liver disease	1.39	0.5–3.83	0.65	0.2002
Charlson index	1.21	1.11–1.33	0.001	0.1723
Organ transplant	0.61	0.2–1.87	0.08	0.1965
Immunosuppressive therapy	0.7	0.28–1.74	0.40	0.1962
Corticosteroid therapy	1.09	0.45–2.65	0.21	0.1964
Bedridden functional capacity	3.13	1.83–5.37	< 0.001	0.1683
Chronic renal impairment	1.16	0.67–2.03	0.23	0.1817
UTI within 1 year	0.77	0.42–1.41	0.11	0.1878
Antibiotic within 30 days	0.63	0.32–1.23	0.09	0.1869
Acquisition of cUTI in a medical care facility	2.42	1.41–4.15	0.001	0.1677
Urinary retention	0.75	0.39–1.44	0.78	0.2149
Neurogenic bladder	1.51	0.6–3.79	0.39	0.2001
Obstructive uropathy	0.46	0.23–0.91	0.05	0.1905
Severe sepsis/ septic shock	11.7	6.18–22.14	< 0.001	0.3077
Polymicrobial infection	1.16	0.63–2.15	0.28	0.1947
MDR-GNB	1.13	0.66–1.93	0.69	0.1887
Urgent admission	1.15	0.55–2.39	0.92	0.2006
Admission reason: UTI	0.28	0.16–0.5	< 0.001	0.1683
Admission from a medical care facility	2.49	1.47–4.19	0.001	0.2014
*Escherichia coli*	0.67	0.42–1.1	0.09	0.1946
*Klebsiella pneumoniae*	1.05	0.56–1.96	0.95	0.1989
*Pseudomonas aeruginosa*	0.7	0.31–1.58	0.41	0.1998
Enterococcus	0.9	0.38–2.15	0.91	0.1999
*Proteus mirabilis*	1.58	0.75–3.32	0.13	0.1947
Urinary catheter as infection source	2.56	1.52–4.32	< 0.001	0.1471

*OR* odds ratio, *95%CI* 95% confidence interval, *ICC* intra-class correlation, *UTI* urinary tract infection, *cUTI* complicated urinary tract, and *MDR-GNB* Multidrug-resistant Gram-negative bacteria

^a^The ICC (range 0 to 1) expresses the magnitude of clustering. A high ICC (generally higher than 0.1) means that individuals within centers are more similar than between centers

**Table 4:** Adjusted logistic mixed-effects models of predictive factors for 30-day mortality

	Model 1			Model 2			Model 3		
	All cohort			Admitted for UTI			Admitted for other condition		
	OR	95% CI	p	OR	95% CI	p	OR	95% CI	*p*
Fixed parts									
(Intercept)	0.08	0.04–0.16	**< 0.001**	0.02	0.01–0.08	**< 0.001**	0.07	0.03–0.18	**< 0.001**
CA-UTI	1.40	0.77–2.54	0.269	1.62	0.63–4.15	0.314	1.24	0.54–2.81	0.610
Male gender	0.53	0.31–0.90	**0.019**	0.31	0.12–0.80	**0.016**	0.77	0.40–1.51	0.450
Age	1.57	1.12–2.19	**0.009**	1.69	0.93–3.05	0.084	1.57	1.03–2.39	**0.036**
Haematologic malignancy	6.08	1.84–20.07	**0.003**	2.33	0.19–29.45	0.512	14.82	2.83–77.61	**0.001**
Charlson score	1.45	1.12–1.88	**0.005**	1.28	0.83–1.96	0.257	1.64	1.16–2.33	**0.005**
Bedridden functional capacity	2.48	1.39–4.43	**0.002**	3.10	1.13–8.50	**0.028**	2.00	0.94–4.28	**0.073**
Acquisition in a medical care facility	1.73	0.93–3.23	0.085	1.58	0.63–3.95	0.327	1.71	0.69–4.25	0.247
Admission reason: other condition than UTI	2.70	1.40–5.00	**0.003**						
Random effects									
τ_00_	0.37			0.96			0.24		
ICC	0.1			0.23			0.09		
Observations	807			474			333		
Marginal R^2^/Conditional R^2^	0.278/0.350			0.210/0.389			0.223/0.292		

*UTI* urinary tract infection, *OR* odds ratio, *95%CI* 95% confidence interval, *CA-UTI* catheter-associated urinary tract infection, *ICC* intra-class correlation

Microbiology, including the presence of MDR-GNB, had no effect on 30-day mortality. The sub-analysis of 668 cases in which we could assess the adequacy of empiric antibiotic treatment failed to show that this variable affected the 30-day mortality (Additional file 1). The factors predictive of 30-day mortality in patients with CA-UTI in the entire cohort were as follows: male gender (OR 0.53; 95% CI 0.31–0.90) as protective factor and age (OR per year 1.57; 95% CI 1.12–2.19), having a haematological malignancy (OR 6.08; 95% CI 1.84–20.07), Charlson index (OR 1.45 per point; 95% CI 1.12–1.88), being bedridden (OR 2.48; 95% CI 1.39–4.43) and being admitted for other condition than UTI (OR 2.70; 95% CI 01.40–5.00) as risk factors. In the subgroup of patients admitted for UTI only male gender and being bedridden were independent predictors of mortality, while in the subgroup of patients admitted for other conditions than UTI predictive factors were age, having a haematological malignancy, Charlson score and being functionally bedridden.

#### Secondary outcomes

Patients with CA-UTI had prolonged overall length of hospital stay (median 14 [IQR 7–27] days vs 8 [IQR 5–14] days, *p* < 0.001) than patients with other cUTI, nevertheless, when stratifying by admission reason this increment was at the expense of the subgroup of patients admitted due to other conditions than UTI (median 20 [IQR 12–30] days in CA-UTI vs 12 [IQR 8–21] days in other cUTI, p < 0.001). The subgroup of patients admitted for UTI showed similar length of stay regardless of the source of UTI (CA-UTI vs others), as shown in Table 5. There were no differences in other outcome variables between groups.

**Table 5:** Clinical outcomes in the CA-UTI and other-cUTI groups

Outcomes	CA-UTI (*n* = 341)	Other cUTI (*n* = 466)	*p*
LOS of patients admitted for UTI (*n* = 474)^a^	8 (5–19)	7 (5–12)	0.226
LOS of patients admitted for other condition (*n* = 333)^a^	20 (12–30)	12 (8–21)	< 0.001
Improvement of symptoms at 5–7 days of treatment, *n* (%)	238 (70)	343 (73.6)	0.26
Recurrence of symptoms at 30 days, *n* (%)	28 (8.3)	42 (9.1)	0.69
Readmission at 60 days of discharge, *n* (%)	56 (16.4)	91 (19.5)	0.25

*CA-UTI* catheter-associated urinary tract infection, *LOS* length of stay, *cUTI* complicated urinary tract infection, *IQR* interquartile range

^a^Length of stay calculated from diagnosis of cUTI to discharge from hospital

## Discussion

In this multicentre, multinational, retrospective cohort study we observed that CA-UTI was the most frequent source of cUTI, typically involving older males with more comorbidities and MDR-GNB. The 30-day mortality of the CA-UTI group was higher than that of the other-cUTI group, but after adjusting for confounders, CA-UTI was not independently associated with 30-day mortality.

The present study included a large and recent cohort of patients with cUTI. The profile of patients with CA-UTI is consistent with that reported in previous studies [2, 19], with these patients frequently having polymicrobial infections and MDR-GNB other than *E. coli* [20]. The repeated antibiotic courses and the healthcare environment to which these patients are exposed both increase the risk of acquiring MDR-GNB strains.

Our CA-UTI group had higher 30-day mortality rates than our other-cUTI group. However, the adjusted models of mortality in the entire cohort and in the subgroups of patients according to admission reason accounting for variation between hospitals and confounding factors did not show an association between 30-day mortality and presence of CA-UTI. Nevertheless, OR of CA-UTI in patients admitted for other condition than UTI was lower than of patients admitted due to UTI, indicating that in the latter group comorbidities had the greatest impact on mortality. The presence of MDR-GNB or adequate empirical antibiotic treatment did not influence mortality in patients with CA-UTI. It should be noted that patients with severe sepsis or septic shock were excluded from these analyses because this factor strongly affects mortality and would have precluded evaluation of the effect of CA-UTI.

The relationship between CA-UTI and mortality risk in previous studies varies significantly. One study of trauma patients admitted to a single institution showed a significant association between mortality and CA-UTI, although this was mostly observed to be associated with increasing age [11]. Another study involving patients following cardiac surgery showed 30-day mortalities of 10.9 and 3.2% in patients who developed CA-UTIs or other cUTIs, but none of these deaths were directly attributed to the cUTI. Multivariate analysis discarded an association between developing a CA-UTI and mortality. Nonetheless, the authors argued that efforts to reduce CA-UTI would be worthwhile in this population by improving their management. Similar results were found in a study in which patients with CA-UTI in intensive care unit and general ward settings both had significantly higher mortality than those without CA-UTI; but, these results did not remain significant after adjustment [10].

Chant et al. [21] performed a meta-analysis of 11 observational case-control studies assessing the risk of mortality associated with CA-UTI among critically ill patients. They found no association between CA-UTI and 30-day mortality after adjusting for confounders, but did find an association between CA-UTI and length of stay, which was increased. We observed longer stays after UTI diagnosis among patients with CA-UTI than among patients without CA-UTI, mainly at the expense of those admitted for other condition than UTI. It’s probable that the greater comorbidities in these patients lead to a more protracted clinical course of UTI.

As previously reported, we showed that administering adequate empiric antibiotic treatment to patients with CA-UTI made no difference to outcomes. When we also consider the role of MDR-GNB in this population, it has been suggested that treatment should be delayed until the results of sensitivity tests are available [22]. However, empiric treatment should still be initiated as soon as possible to avoid adverse outcomes in patients presenting with severe sepsis or septic shock.

The present study has a number of limitations that should be acknowledged. Notably, as with any retrospective observational study, there is a potential for residual confounding from factors that can influence 30-day mortality but that were not evaluated. Nevertheless, our results are reinforced by the large-scale multi-centre design and the fact that we considered possible differences in outcomes between hospitals. By including patients with different characteristics and from different countries increases the generalisability of our data. In addition, the presence of competing events that could modify outcomes was ruled out.

## Conclusions

Patients with CA-UTI tend to be older and to have greater morbidity and mortality than patients with cUTI of other sources. Despite this, the 30-day mortality does not appear to be directly related to the presence of CA-UTI.

## Supplementary information

**Additional file 1.** Antimicrobial resistance profile of Gram-negative bacteria and adjusted logistic mixed-effects model of predictive factors of 30-day mortality (with data about adequacy of empiric antibiotic treatment) in patients with catheter-associated urinary tract infection

## Data Availability

The datasets used and/or analysed during the current study are available from the corresponding author on reasonable request.

## Abbreviations

CA-UTI: catheter-associated urinary tract infections
CFU: colony-forming units
CI: confidence interval
cUTI: complicated urinary tract infections
eCRF: electronic case report form
ICC: intra-class correlation
LTCF: long-term care facilities
MDR: multidrug-resistance
MDR-GNB: multidrug-resistant Gram-negative bacteria
OR: Odds ratio
other-cUTI: cUTI of other source
US: United States
UTI: urinary tract infections
XDR: Extensive drug-resistance
