# Supplementary material for: Clinical outcomes of hospitalised patients with catheter-associated urinary tract infection in countries with a high rate of multidrug-resistance: the COMBACTE-MAGNET RESCUING study
Source: Antimicrob Resist Infect Control. 2019 Dec 3;8:198. doi: 10.1186/s13756-019-0656-6 (PMC6892205; doi:10.1186/s13756-019-0656-6)
Supplement: Supplementary file 1 — Additional file 1. Antimicrobial resistance profile of Gram-negative bacteria and adjusted logistic mixed-effects model of predictive factors of 30-day mortality (with data about adequacy of empiric antibiotic treatment) in patients with catheter-associated urinary tract infection [file 13756_2019_656_MOESM1_ESM.docx]

**Antimicrobial resistance profile of Gram-negative bacteria in patients with catheter-associated urinary tract infection (n = 318)**

| **Antibiotic** | **Resistance (n)** | **(%)** |
| --- | --- | --- |
| Third and fourth generation cephalosporins | 137 | 43.1 |
| Aminoglycosides | 117 | 36.8 |
| Piperacillin-tazobactam | 73 | 22.9 |
| Fluoroquinolones | 174 | 54.7 |
| Carbapenems | 62 | 19.5 |
| Multidrug-resistance | 117 | 36.8 |
| Extensively-drug resistance | 26 | 8.17 |

**Adjusted logistic mixed-effects model of predictive factors of 30-day mortality in patients with catheter-associated urinary tract infection (with data about adequacy of empiric antibiotic treatment) (n = 668)**

|  | **OR** | **95%CI** | **p** |
| --- | --- | --- | --- |
| **Fixed parts** |  | | |
| (Intercept) | 0.08 | 0.03-0.20 | <0.001 |
| CA-UTI | 1.24 | 0.64-2.40 | 0.518 |
| Male gender | 0.42 | 0.23-0.76 | **0.004** |
| Age | 1.67 | 1.14-2.44 | **0.009** |
| Haematologic malignancy | 12.41 | 2.77.55.5 | **0.001** |
| Charlson score | 1.67 | 1.26-2.21 | **<0.001** |
| Bedridden functional capacity | 2.85 | 1.49-5.46 | **0.002** |
| Acquisition in a medical care facility | 1.84 | 0.92-3.69 | 0.085 |
| Admission reason: other condition than UTI | 3.03 | 1.53-6.25 | **0.001** |
| Adequate empiric ATB treatment | 1.16 | 0.62-2.15 | 0.647 |
| **Random parts** | | | |
| τ_00_ | 0.29 | | |
| ICC | 0.08 | | |
| Observations | 668 | | |
| Marginal R^2^/Conditional R^2^ | 0.337/0.390 | | |

OR: Odds ratio, 95%CI: 95% interquartile range, CA-UTI: catheter-associated urinary tract infection, ATB: antibiotic, ICC: intra-class correlation
